# International Medical Congress

**Published:** 1881-06-20

**Authors:** 


### *INTERNATIONAL MEDICAL CONGRESS.*

It is estimated that 2,000 foreign medical men will attend the International Medical Congress to assemble in London early in the month of August next. The International Medical and Sanitary Exhibition will open at the same time and place.

